# Cell Adhesion and Initial Bone Matrix Deposition on Titanium-Based Implants with Chitosan–Collagen Coatings: An In Vitro Study

**DOI:** 10.3390/ijms24054810

**Published:** 2023-03-02

**Authors:** Francesca Veronesi, Silvia Brogini, Angela De Luca, Davide Bellini, Veronica Casagranda, Milena Fini, Gianluca Giavaresi

**Affiliations:** 1 Surgical Sciences and Technologies, IRCCS Istituto Ortopedico Rizzoli, 40136 Bologna, Italy; 2 Novagenit S.r.l., Mezzolombardo, Via Trento 115, 38017 Trento, Italy; 3 Scientific Direction, IRCCS Istituto Ortopedico Rizzoli, 40136 Bologna, Italy

**Keywords:** bone implants, biomaterials, biocompatibility, Ti-alloy coatings, collagen, chitosan

## Abstract

In orthopedics, titanium (Ti)-alloy implants, are often considered as the first-choice candidates for bone tissue engineering. An appropriate implant coating enhances bone matrix ingrowth and biocompatibility, improving osseointegration. Collagen I (COLL) and chitosan (CS) are largely employed in several different medical applications, for their antibacterial and osteogenic properties. This is the first in vitro study that provides a preliminary comparison between two combinations of COLL/CS coverings for Ti-alloy implants, in terms of cell adhesion, viability, and bone matrix production for probable future use as a bone implant. Through an innovative spraying technique, COLL–CS–COLL and CS–COLL–CS coverings were applied over Ti-alloy (Ti-POR) cylinders. After cytotoxicity evaluations, human bone marrow mesenchymal stem cells (hBMSCs) were seeded onto specimens for 28 days. Cell viability, gene expression, histology, and scanning electron microscopy evaluations were performed. No cytotoxic effects were observed. All cylinders were biocompatible, thus permitting hBMSCs’ proliferation. Furthermore, an initial bone matrix deposition was observed, especially in the presence of the two coatings. Neither of the coatings used interferes with the osteogenic differentiation process of hBMSCs, or with an initial deposition of new bone matrix. This study sets the stage for future, more complex, ex vivo or in vivo studies.

## 1. Introduction

Titanium (Ti) is a quasi-bioinert biomaterial and, in the form of alloys, Ti is the most preferred metal for bone implants, due to its high specific strength, low density, corrosion resistance, and biocompatibility [1,2]. Ti-alloys also show a lower Young’s modulus as compared to others, such as stainless steel or cobalt chrome [3].

Nevertheless, the failure of Ti-based implants is not uncommon, due to toxic outcomes of Ti, as well as to the “stress shielding” effect, a result of the tissue–implant mechanical mismatch [4].

An effective strategy for reducing or eliminating stress shielding, and simultaneously enhancing stable long-term fixation, by means of full bone ingrowth and remodeling, has been the development of open pored metallic structures, through additive manufacturing (AM) techniques [5].

The success or failure of an implant is largely dependent on the extent to which it induces bone matrix ingrowth and integrates into the surrounding bone. The greater the osseointegration, the higher the initial mechanical stability, and the lower the probability of implant loosening, with the formation of fibrous tissue at the interface. In addition to mechanical and topographic characteristics, an appropriate surface modification, such as a proper biocoating, also plays a pivotal role for the success of an implant [6]. The implant surface covering enhances bone matrix ingrowth and biocompatibility, improving the long-term stability between new bone formation and the implant, and reducing the healing time [7,8].

Active biomolecules, such as peptides, collagens, and proteins, or growth factors (GFs), such as bone morphogentic protein-2 (BMP2), are used to increase Ti-based implant biocompatibility and physicochemical performance, leading to an increase in cell responses, bone matrix ingrowth and, thus, osseointegration [9].

Among them, type I collagen (COLL I), due to its hemostatic properties, superior biocompatibility, bioactivity, bioresorbability, and low immunogenicity and antigenicity, has been widely used as a tissue replacement material in several medical applications [10]. COLL I, the most abundant protein in mammals, accounts for nearly 95% of the organic matrix in bone, and induces osteoblast (OB) activities, such as proliferation, differentiation, migration, and secretion of extracellular matrix (ECM), providing a suitable environment for bone formation [11].

In preclinical and clinical studies, a significant increase in bone growth and bone-to-implant contact (BIC) around Ti implants has been observed after surface treatment with COLL I [10,12,13,14,15,16,17].

Chitosan (CS), a deacetylated derivative of the natural polysaccharide chitin, is widely used in several biomedical applications [18,19,20,21,22,23,24,25]. It is a bioactive natural alkaline polysaccharide, positively charged, biocompatible, biodegradable, inexpensive, nontoxic, with wound-healing activity, and antibacterial and antimicrobial properties. CS is comparable to bone and cartilage ECM in its composition and chemical structure, and its products are easily metabolized inside the human body by some enzymes [22]. Some studies have shown that scaffolds with layers of CS display improved implant biocompatibility, osteoconductivity, and bone regeneration, and induce OBs and MSC proliferation and neovascularization. Finally, CS also increases cell adhesion and protein adsorption on Ti-alloy implants, consequently improving the osseointegration [18,19,25].

In the literature, there are still very few studies that fabricated composite CS/COLL coverings for bone implants. CS/COLL/hydroxyapatite nanofibers, coated with platelet-rich plasma (PRP), affected the osteogenic differentiation of OBs [26], and a bilayered COLL/CS membrane induced significant expression of osteogenic genes in mesenchymal stem cells (MSCs), and bone formation with no inflammation, in calvarial defects, in an animal model [27].

No study in the literature has evaluated the osteogenic potential and matrix deposition of a Ti-alloy, coated with three COLL/CS multilayers. Recently, we have published a study, in which Ti-alloy (Ti-6Al-4V) (Ti-POR) cylinders, coated or not with COLL I, were evaluated for their osseointegration ability, in an ex vivo study, developed by culturing rabbit cortical bone segments with these cylinders. It was observed that COLL I improved osseointegration and bone growth of Ti-POR [28].

The present study is an upgrade of the previous one [28], where the same Ti-POR samples were covered with two types of multilayered coatings: one made of collagen-chitosan-collagen (COLL–CS–COLL) and the other of chitosan–collagen–chitosan (CS–COLL–CS). Since the antibacterial and antimicrobial properties of CS-coated implants have already been tested, the aim of this study was to observe the ability of these two types of coating to induce cell adhesion and proliferation and form bone matrix. To reach this aim, after cytotoxicity evaluations, human bone marrow MSCs (hBMSCs) were seeded onto Ti-POR specimens, and coated or not with COLL–CS–COLL or CS–COLL–CS coatings for 48 h, and 14 and 28 days. Cell adhesion, proliferation, and osteogenic gene expression, associated with histological and scanning electron microscope (SEM) analyses, were performed.

## 2. Results

### 2.1. Phosphate Buffer Release Test

**Figure 1:**
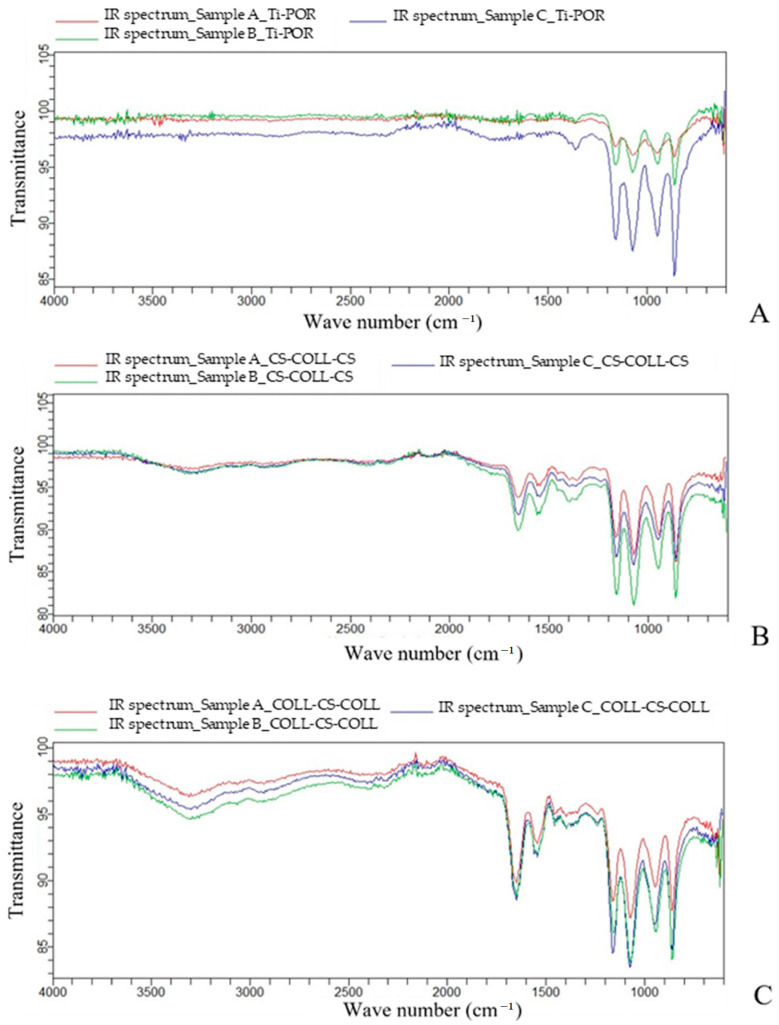
IR spectra overlay of Ti-POR (**A**), CS–COLL–CS (**B**), COLL–CS–COLL (**C**). In red, blue, and green, the IR spectra of three different samples (n = 3) for each type of specimen.

Figure 1 shows the overlays of the IR spectra of Ti-POR, COLL–CS–COLL, and CS–COLL–CS specimens. In the Ti-POR spectra (Figure 1A), the peaks relating to the salts present in PBS were observed between 1200 and 850 cm^−1^, as expected. On the other hand, as regards the coated specimens, in addition to the salt peaks, the characteristic peaks of COLL and CS, between 1650 and 1550 cm^−1^, were present (Figure 1B,C). The solution containing the COLL–CS–COLL specimen showed these peaks in a more marked way, suggesting a slightly higher release of material than the CS–COLL–CS samples (Figure 1B,C). 

### 2.2. Cytotoxicity Results

**Figure 2:**
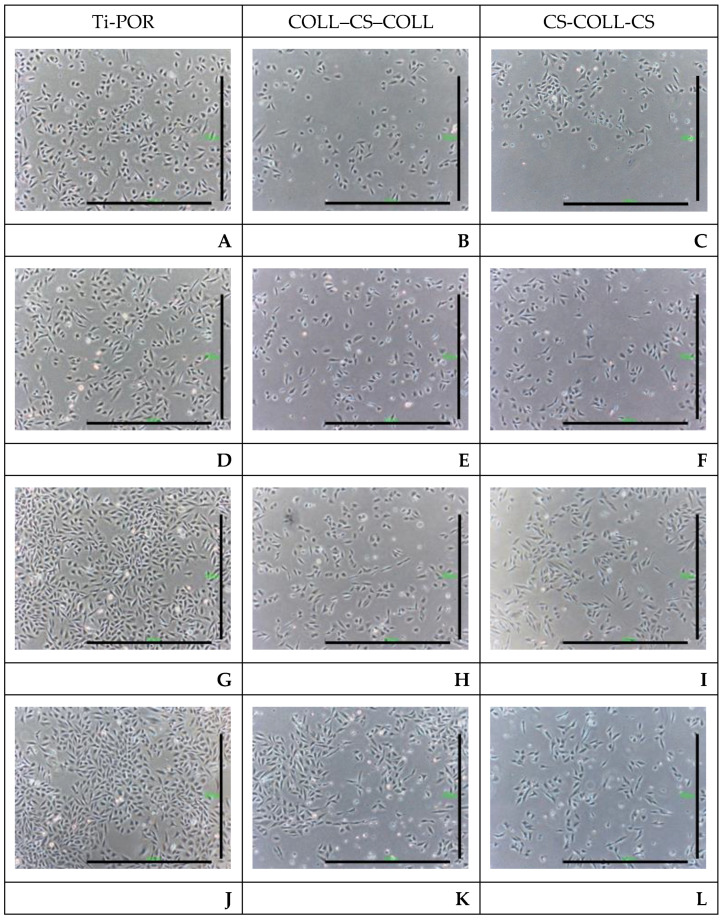
Images of Saos-2 cells cultured in the presence of Ti-POR (n = 2), COLL–CS–COLL (n = 2), and CS–COLL–CS (n = 2) samples, for 6 (**A**–**C**), 24 (**D**–**F**), 48 (**G**–**I**), and 72 h (**J**–**L**), in standard culture conditions. 4X magnification. Bar = 1000 µm.

Figure 2 shows representative microscope images of Saos-2 cells in the presence of Ti-POR, COLL–CS–COLL, and CS–COLL–CS specimens. It was observed that Saos-2 cells in direct contact with Ti-POR specimens maintained their typical morphology over 72 h of culture, and had an increasing proliferation. The Saos-2 cells in contact with COLL–CS–COLL and CS–COLL–CS samples had a slower proliferation than those cultured in the presence of Ti-POR, and mainly maintained their typical morphology, and some cells acquired an elongated shape.

### 2.3. hBMSC Viability and Gene Expression Results

**Figure 3:**
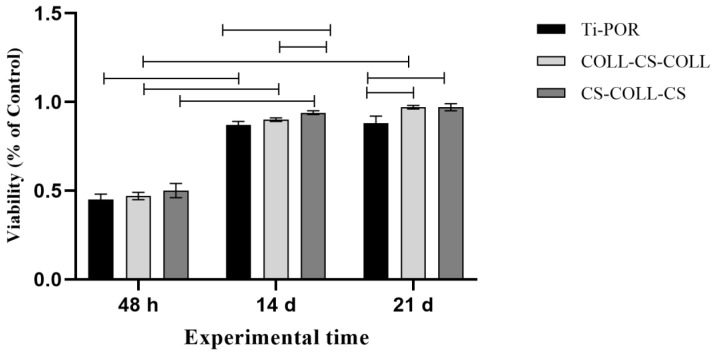
hBMSC viability on Ti-POR (n = 5), COLL–CS–COLL (n = 5), and CS–COLL–CS (n = 5) after 48 h, and 14 and 28 days of culture. Black lines above the histograms have a significance of *p* < 0.05.

**Figure 4:**
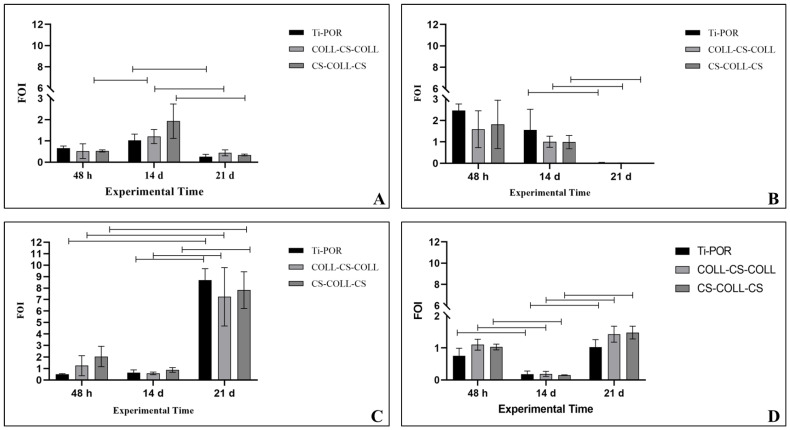
hBMSC gene expression on Ti-POR (n = 5), COLL–CS–COLL (n = 5) and CS–COLL–CS (n = 5) after 48 h, and 14 and 28 days of culture: RUNX2 (**A**), SP7 (**B**), COL1A1 (**C**), ALPL (**D**). Mean ± SD, Mann–Whitney U test (*p* < 0.05). Black lines above the histograms have a significance of *p* < 0.05.

No significant differences among the types of specimens were observed, in the viability of hBMSCs after 48 h of culture. After 14 days of culture, CS–COLL–CS showed significantly higher hBMSC viability than COLL–CS–COLL and Ti-POR (*p* < 0.05). After 28 days of culture, both CS–COLL–CS and COLL–CS–COLL induced significantly higher cell viability than Ti-POR (*p* < 0.05). Over time, the cell viability on COLL–CS–COLL significantly increased (*p* < 0.05), while for CS–COLL–CS and Ti-POR, cell viability showed a significant increase from 48 h to 14 days (*p* < 0.05) (Figure 3). In Figure 4, the results of RUNX2, SP7, COLA1, and ALPL gene expression are shown. As regards RUNX2, for all the samples, its expression was very low (under 1 value), and no significant differences were observed among samples at 48 h and 28 days of culture. At 14 days, COLL–CS–COLL significantly increased RUNX2 expression, in comparison to 48 h and 21 days (*p* < 0.05), and CS–COLL–CS and Ti-POR, in comparison to 21 days (*p* < 0.05) (Figure 4A). SP7 was not expressed after 21 days of culture, and did not show statistically significant differences among samples at all experimental times. For all samples, its expression significantly decreased from 14 to 28 days of culture (*p* < 0.05) (Figure 4B). On the contrary, COL1A1 showed a very low expression (below 1 value) at 48 h and 14 days, up to 28 days of culture, when its expression was significantly higher than at the other experimental times, for all the samples (*p* < 0.05). No significant differences were observed among the samples (Figure 4C). As regards ALPL expression, for all specimens, its expression was significantly higher at 48 h and 28 days of culture in comparison to 14 days (*p* < 0.05), when its expression was almost absent. No significant differences were observed among specimens for any of the experimental times (Figure 4D).

### 2.4. Histology and BS-SEM Results

**Figure 5:**
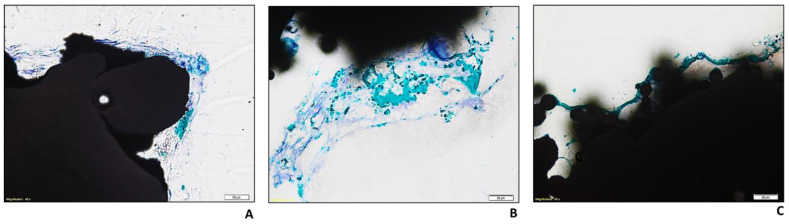
Histological images of Ti-POR (**A**), COLL–CS–COLL (**B**), and CS–COLL–CS (**C**) cultured with hBMSCs at 28 days. Toluidine blue/Fast green staining. Magnification 40X (scale bar = 20 µm).

For Ti-POR, no matrix deposition was observed at 48 h. It gradually increased, starting from 14 days, and it was observable both on the surface and within the cylinder. At higher magnification, an initial mineralization of the matrix, colored in green after Toluidine blue/Fast green staining, can already be seen at 14 days, which becomes more evident at the longest experimental time of 28 days (Figure 5A).

**Figure 6:**
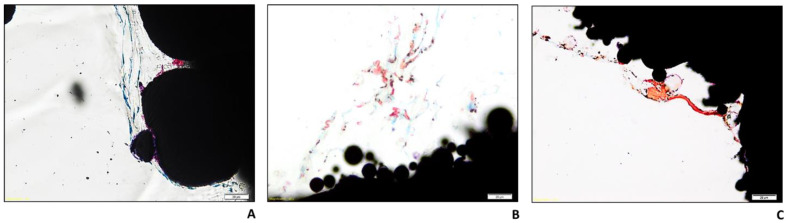
Histological images of Ti-POR (**A**), COLL–CS–COLL (**B**), and CS–COLL–CS (**C**) cultured with hBMSCs at 28 days. Stevenel’s blue/Picrofucsin staining. Magnification 40X (scale bar = 20 µm).

As for the COLL–CS–COLL and CS–COLL–CS samples, the presence of a filamentous reticular structure, reactive to the Toluidine blue dye, can be seen, both on the surface and inside the material, regardless of the experimental time. After 28 days, for both types of coatings, the presence of areas reactive to the Fast green dye was observed, both on the surface of the materials and along the filaments described above (Figure 5B,C). Compared to Ti-POR, these areas are clearer, larger, as well as more easily identifiable, and uniformly distributed along the entire perimeter of the samples. The staining with Stevenel’s blue and Picrofuchsin confirmed the presence of areas with different degrees of mineralization in the samples, at 28 days (Figure 6).

**Figure 7:**
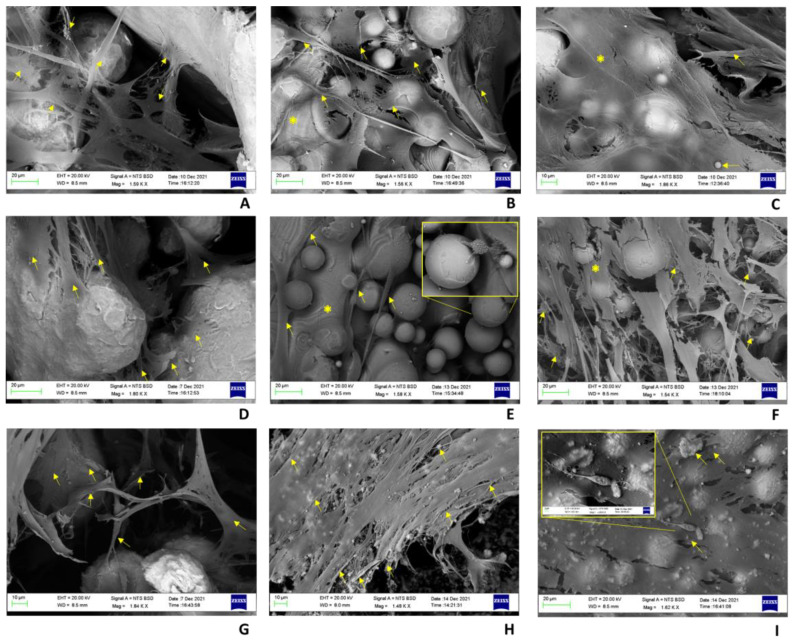
BS-SEM images of Ti-POR (**A**,**D**,**G**), COLL–CS–COLL (**B**,**E**,**H**), and CS–COLL–CS (**C**,**F**,**I**) cultured with hBMSCs for 48 h (**A**–**C**), 14 days (**D**–**F**), and 28 days (**G**–**I**). 1.59–1.84KX magnification (**A**,**D**,**G**); 1.44–1.56KX magnification (**B**,**F**,**H**); 1.58−2.66KX magnification (**E**); 1.23–1.86KX magnification (**C**); 1.62–1.64KX magnification (**I**). hBMSCs are indicated with arrows, and the two layers, COLL–CS–COLL or CS–COLL–CS, are indicated with asterisks.

The analysis of the BS-SEM images showed the degree of colonization of the specimens and the de novo matrix production by the hBMSCs, confirming the histological data (Figure 7). On Ti-POR, already at 48 h, the hBMSCs were clearly visible, and the colonization increased over time, both in terms of the number of cells visible under high magnification and in the newly deposited matrix, which progressively covered the surface of the material.

The images of the COLL–CS–COLL samples showed the amorphous matrix covering the material, and at higher magnifications the cells were visible, which together with the matrix produced, formed a further layer enveloping the COLL–CS–COLL multilayer coating. At 28 days, cells were visible at intermediate magnifications, indicating increased cell growth and matrix deposition by cells, which made it more difficult to discriminate individual cells, except at high magnifications (Figure 7). Similarly, for CS–COLL–CS, cells colonizing the material were clearly visible at low and intermediate magnifications. At 28 days the hBMSC colonization was easily visible, even at intermediate magnifications, forming a layer of cells and a new matrix, deposited on the CS–COLL–CS multilayer (Figure 7).

## 3. Discussion

The present in vitro study evaluated the cell adhesion and bone matrix formation ability of two new multilayered coatings of a Ti-alloy implant (Ti-POR), for further employment in the orthopedic field. The study showed that both Ti-alloy coatings, made of composite COLL/CS layers, were cytocompatible, improving hBMSC adhesion and subsequent colonization from the shortest experimental time, of 48 h. Furthermore, a progressive increase in matrix deposition was observed, and an initial mineralization started from 28 days of culture.

For this purpose, hBMSCs were chosen as cells to be seeded onto samples, to evaluate their osteogenic commitment and bone matrix production ability up to 28 days of culture. Cell viability and gene expression analyses were performed, in association with histological and SEM evaluations.

Ti-POR samples, manufactured using the additive manufacturing technique of EBM, on which the coverings were applied, were the same used in two previous ex vivo and in vivo studies [11,28]. The samples showed a 3D superficial open porous structure of Ti6-Al-4V, interconnected with a solid central metallic core. The superficial porous layer (1 mm thick) had an average pore diameter of 700 μm. This pore dimension falls in the range 300–1000 μm, the upper and lower bounds of which are considered to be the minimum and maximum pore dimensions that can promote bone ingrowth [29].

After having prepared the 3D CAD model of the porous cylinders, a dedicated software sliced the 3D model into 50 μm layer thicknesses [28].

Surface composition plays an essential role in determining the initial cellular and molecular response of cells that meet the implant. In the literature, the biocompatible and antifungal properties of CS are well recognized, and together with its high antimicrobial and biodegradability characteristics, it and its derivatives have already been employed in several medical applications [30]. It is often used as a dressing material, in the manufacture of drugs as a controlled-release active substance carrier, or in tissue engineering involving soft tissues, nerves, cartilage, bones, or arteries [31,32]. Furthermore, CS is also reported to be an excellent material for growing OBs, such as glycosaminoglycans and hyaluronic acid, thanks to its structural characteristics [33]. Given the promising results of CS for implant applications, its performance and capabilities need to be further assessed and developed for bone. On the other hand, several studies have already tested the osteogenic potential of COLL, the most abundant protein of bone ECM, in numerous orthopedic and dental applications [11,12,14,16,17,28]. For the above-mentioned reasons, in the present study, CS and COLL were evaluated in combination, as an innovative covering of Ti-alloy samples.

Through an innovative spraying technique, the two types of multilayered coverings were fabricated, made by COLL–CS–COLL or CS–COLL–CS trilayers. The PBS release test evidenced the presence of the characteristic peaks of COLL and CS between 1650 and 1550 cm^−1^, in association with the peaks of PBS salt.

A first evaluation of cytotoxicity, showed that Saos-2 cells reduced their proliferation and acquired an elongated shape in the presence of both COLL–CS coatings, indicating that cells started to differentiate towards more mature cells and started to produce bone matrix. As regards hBMSCs’ viability, the results showed that, up to 28 days of culture cells remained vital, with a significant increase from 48 h to 14 days of culture, and then maintaining stable growth from 14 to 28 days. In addition, both coated specimens induced a higher cell proliferation than the non-coated one, at 14 and 28 days.

For gene expression analysis we chose the most representative osteogenic differentiation genes, that encode for both early and late bone formation markers. Among them, *RUNX2* and *SP7* genes encode for transcription factors that are considered early osteogenic markers, showing an essential role in the development of OBs, driving the differentiation of BMSCs into OBs and eventually osteocytes with their expression, that decreases when BMSCs differentiate towards OBs [34,35]. *COLA1* is a gene that encodes for the pro-alpha1 chains of COLL I, and is considered a late marker of osteogenesis. It is a fibril-forming collagen found in most connective tissues, abundant especially in bone [36]. Finally, *ALPL* encodes for ALP, an early marker of OB differentiation, which is elevated during osteodifferentiation of MSCs.

In the present study, all coated or not coated specimens showed the same gene expression trend at all experimental times, without observing statistically significant differences among the specimens. This result underlined that all samples, analyzed in this study, induced a similar osteogenic hBMSC differentiation. However, differences in gene expression were highlighted over time. A peak of *RUNX2* expression was detected starting from 14 days of culture and it significantly decreased at 28 days of culture. On the contrary, major levels in the expression of *SP7* were detected at 48 h and the declined at 28 days of culture, at which time no gene activity was observed. Hence, this shift of expression probably determines a later up-regulation of the genes directly regulated by *RUNX2*, such as *ALPL* that showed an opposite trend respect *RUNX2* expression. In literature it was observed that *ALPL* level expression increased after two days of culture, decreased at the intermediate time of 14 days and subsequently upregulated again at 28 days, when matrix mineralization largely covered the culture vessels [37]. On the other hand, *COL1A1* expression was significantly higher at 28 days of culture than at 48 h and 14 days, underlining its late marker nature.

To appreciate the ability of the specimens to induce cell adhesion and bone matrix formation, even at high magnification, we decided to use histology and SEM. After 48 h of culture, in Ti-POR samples no matrix deposition was observed, while, the other two coated specimens, both COLL–CS–COLL and CS-COLL-CS, induced filamentous reticular structure already at 48 h of culture.

Ti-POR gradually increased matrix deposition starting from 14 days of culture, both at the surface and inside the implant. Also, COLL–CS–COLL and CS–COLL–CS specimens increased the production of mineralized matrix on surface and inside the implants, but these areas are larger than those present on Ti-POR material. To better confirmed the presence of these matrix areas, Stevenel’s blue/Picrofuchsin histological staining was performed. This staining confirmed the presence of areas with different degrees of mineralization in the samples at 28 days. The Stevenel’s blue stain shows cells and extracellular structures in various shades of blue (except for mineralized tissues). Counterstaining with Picrofuchsin makes collagen fibers a bluish-green color, bone orange or purple, and osteoid yellow-green [38].

The use of BS-SEM helped to appreciate and confirmed at higher magnification what histology already did, and that is the degree of cell colonization and the de novo bone matrix production. After 48 h of culture, Ti-POR was colonized by hBMSCs and both cell colonization and new matrix deposition increased over time. Also, COLL–CS–COLL specimen and similarly CS–COLL–CSone were covered by an amorphous matrix with cells that created a layer of matrix over the coating, with an increase in cell growth and matrix deposition from 48 h to 28 days of culture. 

The results of the present in vitro study are certainly preliminary, but are a first step to observing whether new innovative coatings can induce the formation of bone matrix in humans. Obviously, these data will need to be confirmed in subsequent in vivo or ex vivo studies, where it is also possible to evaluate the parameter of osseointegration, in a microenvironment as similar as possible to the physiological condition. Concerning that, the ethical adhesion to the 3R principles requires the progressive decrease and replacement of animal use for preclinical studies [39]. So, to find alternative methods to preclinically evaluate implants, bone matrix formation, and osseointegration, with more advanced methods, able to simulate the clinical condition as much as possible, in a previous study we set up an alternative ex vivo model. This model has been confirmed to be useful in evaluating the osseointegration of implant materials, by culturing cortical bone segments adhered to Ti-POR cylinders, and evaluating bone-to-implant contact (BIC) and new bone formation (nBAr/TAr) after 30, 60, and 90 days of culture. This ex vivo model is seen to reproduce the complex in vivo microenvironment, with a view to reducing the number of animals used in vivo [40].

## 4. Materials and Methods

### 4.1. Preparation of Titanium Specimens (Ti-POR)

The Ti-alloy (Ti-6Al-4V) cylinders (4 mm in diameter and 8 mm in length) were manufactured by Adler Ortho^®^ SPA (Cormano, Milan, Italy), as reported in the previous study [28]. The specific structure and shape of the implants were designed based on a previous virtual Computer-Aided Design (CAD) model. Each implant has an interconnected porous structure of Ti-6Al-4V, with a solid central core, and an external porous (average porosity ∅ = 700 μm) layer, with dimensions like trabecular bone. Metal powders were added layer by layer by electron beam melting (EBM) (ARCAM EBM-GE Additive, Gothenburg, Sweden) on Ti-6Al-4V (Ti-POR). Subsequently, all Ti-POR implants were washed in an ethanol/water (80%/20%) mixture for 48 h, and then left in 100% ethanol for 72 h, before air drying.

### 4.2. Multilayer Coating of Ti-POR (COLL–CS–COLL and CS-COLL-CS)

The bio-layers used in the present work consisted of equine COLL I (Euroresearch S.r.l., Milan, Italy) and CS (HMC 90/200-Heppe Medical Chitosan GmbH, Saale, Germany). Both COLL I and CS were used in fluid form, to cover the Ti surface of Ti-POR, with an innovative spraying technique developed in Novagenit S.r.l. laboratories. It consists in spraying fluidified solution of both COLL I and CS directly onto metal samples through a nebulizer. About 100 g of equine COLL I gel was weighed and fluidified at about 45–50 °C, with a magnetic stirrer (IKA RCT Basic, Staufen Germany), monitoring the temperature with a temperature probe. About 500 mg of CS powder was solubilized in 50 mL of 1.5% acetic acid solution in water.

The formation of each single layer involved:

1. The spraying of fluidized COLL or CS on the surface of the Ti-POR implants;
2. Rapid freezing at −40 °C;
3. Freeze-drying.

In this way, it was possible to create Ti specimens with a triple coating layer: some implants with a coating made of collagen-chitosan-collagen (COLL–CS–COLL) and others with a chitosan–collagen–chitosan coating (CS–COLL–CS).

**Figure 8:**
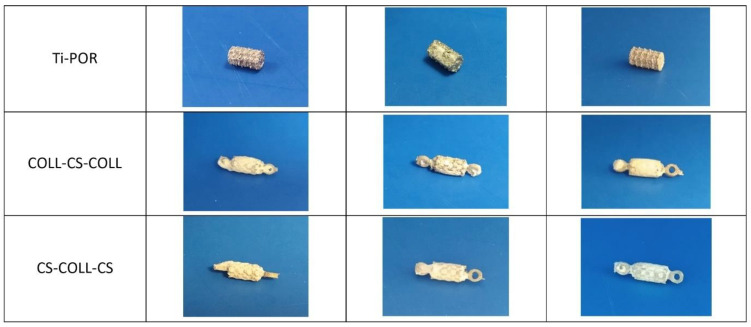
Representative photos of Ti-POR, COLL–CS–COLL and CS–COLL–CS specimens employed in the study.

At the end of the lyophilization of the last layer, all specimens were dried in a vacuum oven at 37 °C for 96 h. Finally, they were individually packaged and sterilized at 25 kGy. Uncoated cylinders (Ti-POR) of the same dimension were used as control substrates. Figure 8 shows the three types of specimens tested in the present study.

The porous and interconnected structure of the specimens does not allow for the measurement of the layers of COLL and CS accurately and homogeneously on the surface of the Ti-POR specimens.

The quantification of both materials anchored on the Ti-POR samples was made by calculating the difference in weight of the specimens before and after the coating treatment.

### 4.3. Phosphate Buffer Release Test

Each of the sterile coated and non-coated specimens were dipped into a well of a 24-well plate, with 2 mL of Dulbecco’s phosphate-buffered saline (PBS) (Sigma-Aldrich, Milan, Italy), to perform the phosphate buffer release test. The evaluations were performed in triplicate for each type of specimen. The 24-well plate was placed in a CO_2_ incubator at 37 °C for 28 days. At this experimental time the specimens were separated from the PBS. The specimens and PBS were freeze-dried separately, and infrared (IR) spectra acquisition (FT-IR Agilent Cary 630 Spectrometer, Agilent Technologies, Santa Clara, CA, USA) was performed on the lyophilized solution, to verify the presence of COLL I and/or CS in the solution. IR spectra of PBS were obtained, to evaluate the release of the multilayers, and therefore the possible presence of COLL I and/or CS.

### 4.4. In Vitro Cytotoxicity Evaluation

Saos-2 cells (commercial source Saos-2 human primary osteogenic sarcoma. Cod. 89050205. Sigma-Aldrich, Milan, Italy) were expanded in T75 flasks (Corning Cell Culture Flask, Sigma-Aldrich Corp., St. Louis, MO, USA) in DMEM/F12, with 10% FBS (Sigma-Aldrich Corp., St. Louis, MO, USA), L-glutamine 2 mM, and penicillin-streptomycin 0.1 mg/mL, in standard culture conditions (at 37 °C, 5% CO_2_, and humidified atmosphere), and the medium was replaced every two–three days. After reaching 85–90% of confluence, 1 × 10^5^ cells/mL were seeded in 6-well plates, with 3 mL of medium per well, and incubated in standard culture conditions for 24 h, to allow the cells to adhere to the wells. Then, the sterile COLL–CS–COLL (n = 2), CS–COLL–CS (n = 2), and Ti-POR (n = 2) specimens were positioned in the wells for 6, 24, 48, and 72 h. At the end of the experimental times, cell proliferation and morphology were qualitatively documented by means of an optical inverted microscope (Eclipse TS100, Inverted Routine Microscope, Nikon Instrument S.p.A., Campi Bisenzio, FI, Italy), and a digital sight camera (Digital Sight DS-L2, Nikon Instrument S.p.A., FI, Italy) at 4X magnification. 

### 4.5. hBMSC Viability

hBMSCs (commercial source, Cod. 492-05a, Cells Applications Inc., San Diego, CA, USA) were expanded in mesenchymal basal medium (MesenCultTM-MSC Basal Medium; STEMCELL Technologies Inc., Vancouver, BC, Canada), completed with the appropriate supplements (MesenCult™ MSC Stimulatory Supplement; STEMCELL Technologies Inc.), 100 U/mL penicillin, and 100 μg/mL streptomycin, (SIGMA, St. Louis, MO, USA) for about 14 days, in T75 flasks (Corning Cell Culture Flask, Sigma-Aldrich Corp., St. Louis, MO, USA), in standard conditions. The medium was replaced every two–three days. An amount of 2.15 × 10^5^ hBMSCs was seeded on each sterile specimen, taking care to uniformly sow the samples: Ti-POR (n = 15), COLL–CS–COLL (n= 15), and CS–COLL–CS (n = 15) cylinders. Test samples were placed in a 12-well plate and incubated for 1 h in standard conditions, to enhance cell adhesion; then 2 mL of fresh basal medium was added. After 24 h of culture, growth medium was completely replaced with osteogenic differentiation medium, composed of completed basal medium supplemented with β-glycerolphosphate (10^−4^ M), ascorbic acid (50 µg/mL), and dexamethasone 10^−7^ M. The medium was replaced every two–three days. After 48 h, and 14 and 28 days, the specimens of each type were analyzed for hBMSC viability.

The viability of hBMSCs was quantified by Alamar blue assay (Invitrogen, Waltham, MA, USA) at each experimental time. Alamar blue dye, mixed with culture medium (1:10 *v*/*v*), was added to hBMSCs, seeded onto the samples, and incubated for 4 h in standard conditions. The amount of fluorescence is proportional to the number of living cells and corresponds to the cells’ metabolic activity. The fluorescent product was quantified at 530ex–590em nm, using a microplate reader (VICTOR X2030, Perkin Elmer, Milano, Italy), and expressed as relative fluorescence units (RFU).

### 4.6. hBMSC Gene Expression

After 48 h, and 14 and 28 days, total RNA was extracted from the cells seeded on the samples, using the commercial RNeasy Mini Kit (Purelink™ RNA miniKit, Ambion by Life Technologies, Carlsbad, CA, USA), quantified by a NANODROP spectrophotometer (NANODROP 2720, Thermal Cycler, Applied Biosystem), and reverse transcribed using the Superscript Vilo cDNA synthesis kit (Life Technologies). Gene expression was evaluated by semiquantitative PCR analysis, using the SYBR green PCR kit (QIAGEN GmbH, Hilden, Germany), in a Light Cycler 2.0 Instrument (Roche Diagnostics, GmbH, Manheim, Germany). Ten nanograms of cDNA were tested in duplicate for each sample. The protocol included a denaturation cycle at 95 °C for 15 min, 25 to 40 cycles of amplification (95 °C for 15″, appropriate annealing temperature for each target, as detailed in Table 1, for 20″, and 72 °C for 20″), and a melting curve analysis to check for amplicon specificity. The mean threshold cycle was determined for each sample and used for the calculation of relative expression using the 2^−ΔΔCt^ method, with GAPDH as the reference gene and Ti-POR as the calibrator [41]. 

### 4.7. Histology 

For histological and SEM analyses, the same processing method was performed on each specimen until the dehydration step in ethanol 70%. Briefly, the samples were fixed in 2.5% glutaraldehyde, in pH 7.4 phosphate buffer 0.1 M, for 1 h, and subsequently dehydrated in a graded ethanol series (30–50–70%) for 15 min each. 

As regards histological analyses, after the preliminary phase, the samples dedicated to histological processing were further dehydrated in successive passages in 95% and 100% alcoholic solutions. Subsequently the samples were infiltrated in two different acrylic-based solutions (Methacrylate; Merck, KGaA, Darmstadt, Germany) until polymerization.

Then, the samples were cut perpendicularly to the major axis of the cylindrical samples, with the Leica SP1600 diamond blade microtome (Leica Microsystems S.r.l., Buccinasco, Italy), obtaining about 15 cross sections for each sample. Consecutive sections were thinned and smoothed using abrasive papers with different granulation, using the Saphir sanding system (Saphir 550, ATM GmbH, Mammelzen, Germany), to obtain a final thickness of the sections of about 50 ± 10 µm. Three of these sections were then subjected to histological staining with Toluidine blue and Fast green, observed under the Olympus BX51 optical microscope (BX51, Olympus Optical Co. Europe GmbH, Hamburg, Germany), and acquired digitally using the Aperio Scanscope digital scanner (CS System, Aperio Technologies, Vista, CA, USA). A further staining with Stevenel’s blue and Picrofucsin, according to Van Gieson, was carried out on another three histological sections of the Ti-POR, COLL–CS–COLL, and CS–COLL–CS samples, only at 28 days.

### 4.8. BS-SEM

After the preliminary phase, samples were further dehydrated for 15 min in 95% ethanol and 100% ethanol, for 1 h. Then they were placed in Hexamethyldisilazane (Sigma Aldrich, Co., St. Louis, MO, USA) for two successive passages of 5 min each, and left to air dry. At the end of these steps the samples were coated in gold for 60 s, using an Agar Sputter Coater (Agar Scientific, Stansted, UK), and mounted on sample holders for SEM, using carbon conductive adhesive discs. The backscattered electronic images (backscattered-BS) were acquired using an SEM, model EVO LS HD (Carl Zeiss S.p.A, Milan, Italy), using the SmartSEM software (version 5.07, Carl Zeiss AG, Oberkochen, Germany), at different magnifications, and in backscattered, to evaluate the adhesion and colonization of the surface by hBMSCs.

### 4.9. Statistical Analysis

Data were analyzed by using the R software, version 4.2.1 [42]. After having checked the non-normal distribution (Shapiro–Wilk test) and non-homogeneity of variance (Levene test) of data, Kruskal–Wallis χ^2^ test, followed by non-parametric Mann–Whitney U test were used for comparisons among groups. Data are reported as mean ± SD, at a significant level of *p* < 0.05. 

## 5. Conclusions

In conclusion, the new two types of coatings made by three layers, COLL–CS–COLL or CS–COLL–CS, are not cytotoxic and induce hBMSC adhesion, sustain their viability, and induce bone matrix deposition on the surface of, and inside, a Ti-alloy implant, without differences between the two coatings. In comparison to the non-coated specimen (Ti-POR), the cell viability results were higher, and bone matrix deposition was observed earlier on the coated specimens. It can therefore be stated that both coatings used do not interfere with the osteogenic differentiation process of hBMSCs and with the deposition of new bone matrix.

## Figures and Tables

**Table 1:** Specifications of primers used for the gene expression analysis.

Gene	Primer Forward	Primer Reverse	Amplicon Length	Annealing Temperature
GAPDH	QuantiTect Primer Assay (Qiagen) Hs_GAPDH_1_SG		95 bp	55 °C
RUNX2	CTTCACAAATCCTCCCCAAGT	AGGCGGTCAGAGAACAAAC	212 bp	60 °C
COL1A1	QuantiTect Primer Assay (Qiagen) Hs_COL1A1_1_SG		118 bp	55 °C
ALPL	QuantiTect Primer Assay (Qiagen) Hs_ALPL_1_SG		110 bp	55 °C
SP7	QuantiTect Primer Assay (Qiagen) Hs_SP7_1_SG		120 bp	55 °C

## Data Availability

The data that support the findings of this study are available from the corresponding author upon reasonable request.
